# Can we identify the prevalence of perinatal mental health using routinely collected health data?: A review of publicly available perinatal mental health data sources in England

**DOI:** 10.1002/lrh2.10374

**Published:** 2023-06-19

**Authors:** Sarah Masefield, Kathryn Willan, Zoe Darwin, Sarah Blower, Chandani Nekitsing, Josie Dickerson

**Affiliations:** ^1^ Department of Health Sciences, Faculty of Sciences University of York York UK; ^2^ Bradford Institute for Health Research Bradford Teaching Hospitals NHS Foundation Trust Bradford UK; ^3^ Department of Allied Health Professions, Sport and Exercise, School of Human and Health Sciences University of Huddersfield Huddersfield UK

**Keywords:** health data systems, inequalities, NHS data, open data, perinatal mental health, system change

## Abstract

**Introduction:**

Perinatal mental health (PMH) conditions affect around one in four women, and may be even higher in women from some ethnic minority groups and those living in low socioeconomic circumstances. Poor PMH causes significant distress and can have lifelong adverse impacts for some children. In England, current prevalence rates are estimated using mental health data of the general population and do not take sociodemographic variance of geographical areas into account. Services cannot plan their capacity and ensure appropriate and timely support using these estimates. Our aim was to see if PMH prevalence rates could be identified using existing publicly available sources of routine health data.

**Methods:**

A review of data sources was completed by searching NHS Digital (now NHS England), Public Health England and other national PMH resources, performing keyword searches online, and research team knowledge of the field. The sources were screened for routine data that could be used to produce prevalence of PMH conditions by sociodemographic variation. Included sources were reviewed for their utility in accessibility, data relevance and technical specification relating to PMH and sociodemographic data items.

**Results:**

We found a PMH data ‘blind spot’ with significant inadequacies in the utility of all identified data sources, making it impossible to provide information on the prevalence of PMH in England and understand variation by sociodemographic differences.

**Conclusions:**

To enhance the utility of publicly available routine data to provide PMH prevalence rates requires improved mandatory PMH data capture in universal services, available publicly via one platform and including assessment outcomes and sociodemographic data.

## 1 INTRODUCTION

Perinatal mental health (PMH) conditions such as depression and anxiety can cause significant distress, interfere with parenting and parent‐child attachment, and have lifelong impacts for children.^1^, ^2^ The long‐term cost to society of each case of PMH difficulties has been estimated at around £74 000 for depression and £35 000 for anxiety, of which almost three‐quarters (72%) relates to the potential adverse impact on the child.^3^

To reduce the potential long‐term impacts of PMH, the World Health Organisation recommends a stepped approach for PMH conditions, using evidence‐based preventative interventions for vulnerable women within usual services, and the provision of targeted services for those with more severe needs.^4^ However, to ensure that women receive appropriate and timely support from services, understanding the prevalence of PMH needs is essential to the capacity planning of these services. Prevalence of PMH conditions across the globe is based on estimates from a relatively small sample of population‐based survey studies, resulting in a wide range of prevalence estimates (between 1 in 10 and 1 in 4), with higher rates reported in low and middle‐income countries.^4^, ^5^, ^6^ In high‐income countries, there is evidence that some ethnic groups and those living in lower socio‐economic circumstances are more likely to experience PMH conditions.^7^ In the UK, support for PMH conditions uses the WHO‐recommended stepped approach within the National Health Service (NHS) with: support for mild to moderate cases provided within usual care by midwives, health visitors and GPs; and moderate to severe cases supported by specialist PMH services, and mother and baby units for the most complex cases. NHS provision is commissioned and planned separately for each nation within the UK, with data also reported separately in each nation. Current estimates of the number of women in England who will need stepped‐support for PMH conditions, are based on prevalence estimates agreed by the Joint Commissioning Panel for Mental Health.^8^ These estimates use evidence from nine studies (not systematic reviews; completed between 1987 and 2011) on the national prevalence estimates of mental health conditions in the general population. The data from these estimates are combined with population estimates from the Office for National Statistics (ONS) and the NHS Digital Quality and Outcomes Framework (QOF) data to provide local prevalence estimates which are shared on the Office for Health Improvement and Disparities (formerly Public Health England) PMH Fingertips dashboard (also known as the PMH Profile). This results in an estimate of 3% prevalence for severe depressive illness and 10%–15% for mild to moderate depressive illness and anxiety.^9^ However, each of these estimates includes a flag to acknowledge concerns about the quality of the data behind the indicator. This is because these estimates use mental health data for the general population, not specifically for women during the perinatal period, and do not take account of variance in conditions experienced by women of different ethnic groups and socioeconomic status.^8^, ^9^ The sociodemographic composition of female perinatal groups varies significantly across the country, which will therefore influence the demand for PMH support and the planning of service capacity required to support women.

In the other UK nations, data availability is also limited for example: in Wales, there are summary statistics only for the number of women who were recorded as having a mental health condition during their initial assessment during pregnancy^10^; in Scotland, mental health data does not include a PMH indicator^11^; and in Northern Ireland prevalence data are only reported on inpatient mental health occurrences.^12^

There is a clear need for more precise PMH prevalence rates. One efficient solution would be to use routinely collected health data (ie, data recorded in electronic health records in community, primary, and secondary care settings as part of administrative and clinical processes). For other conditions, publicly available open data are highly valuable for producing prevalence estimates, and for exploring inequalities relating to social determinants of health and area‐based differences.^13^, ^14^

NHS England has made routine maternity data publicly available since 2015 reiterating their commitment to better data sharing and information in the Maternity Transformation Programme launched in 2016.^15^ The National Institute for Health and Care Excellence (NICE) guidance recommends that during pregnancy and routine postnatal appointments, healthcare professionals ask women about their PMH and use a screening tool, and further assessments where there are concerns.^16^, ^17^ This data could provide in‐depth prevalence data for PMH which reflect area‐based sociodemographic differences.

However, there are concerns that, despite recommendations for routine screening and assessments of PMH, not all women have their problems identified and captured in their health record.^18^, ^19^

There has been little investigation of the utility of routinely collected health data.^20^ As such, an investigation of the prevalence of PMH using routine data is also, necessarily, an investigation of the potential limitations of that data and what it means for the wider understanding of PMH in England.

## 2 QUESTION OF INTEREST

The aim of this study was to explore whether the prevalence of PMH conditions could be identified from publicly available routine health data, and whether these datasets could identify variation in prevalence by key sociodemographic characteristics. To do this, we explored the utility of publicly available data sources of routine PMH data for estimating the prevalence of poor PMH, and exploring potential inequalities in the identification and capture of PMH difficulties at the local (eg, NHS Trust or Clinical Commissioning Group [CCG]) level in England.

## 3 METHODS

We used a review method to identify publicly available national sources of routine health data and screened them for PMH data which could be used to (a) produce prevalence of PMH conditions; (b) produce prevalence based on local area sociodemographic variation.

Data sources were identified by searching the NHS Digital (now part of NHS England) datasets,^21^ PMH data catalogue,^22^ and national PMH guidance documents,^17^, ^23^ performing keyword searches online (‘PMH data’, ‘maternal mental health data’, ‘maternity data’, ‘data for the perinatal period’), and research team knowledge of local and national PMH data contexts.

Data sources were eligible for the review if they were: publicly available (defined as open access online, not requiring a request or payment), contained routine health data for England within the perinatal period (defined as data recorded in health records by NHS and other healthcare professionals working within the perinatal time frame); and available between April and August 2022 (when the search was conducted). We distinguished data sources from data sets, with a data source defined as the platform via which a data set is publicly accessed. Whilst the underlying data used in these sources may be available on request, our focus is on publicly available data sources to allow us to explore what expedient and easily accessible PMH data were available to anyone, and what prevalence estimates could be produced from them.

The key criteria, established by the research team based on their experience of using publicly available data sources, and used to assess the utility of the data sources were:

1. accessibility – access to recent aggregate data (based on individual level data) via an interactive dashboard (allowing users to interact with data by displaying and analysing relevant data items);
2. data relevance – whether the data items in the dataset made direct reference to the perinatal period;
3. technical specification – availability of and insight provided by any data set technical specification documents or flags for data quality issues indicated within the data source.

## 4 RESULTS

**FIGURE 1:**
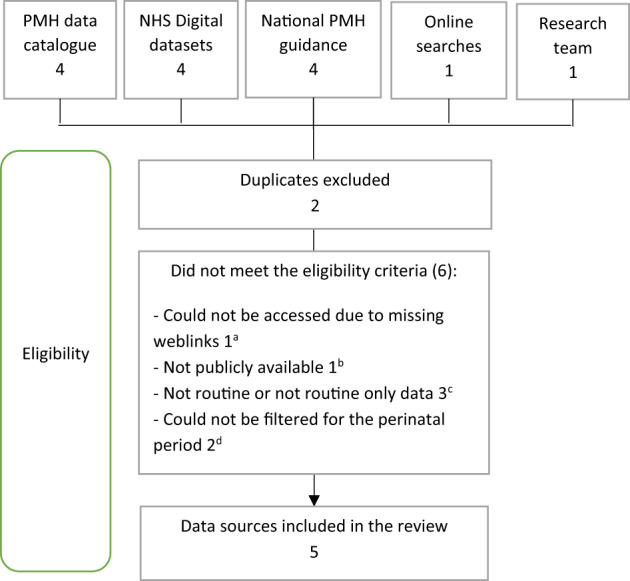
Flow diagram of the eligibility and screening process. ^a^National Child and Maternal (ChiMat) Health needs assessment report.^15^ ^b^NHS Benchmarking Network (also did not currently include PMH data despite being identified as a PMH data source in the PMH data catalogue).^16^ ^c^National enquiry into maternal deaths (MBRRACE‐UK)^17^ (includes data from staff caring for the women concerned, coroners, procurators fiscal and media reports and not fully relevant as focus on maternal deaths due to psychiatric causes); Maternity Services Survey (presents data on self‐reported PMH conditions for a sample of women)^18^; PMH Fingertips (not routine data; not specific data for the perinatal period (presents ‘the likely number of women who are affected by particular mental health conditions’).^6^ ^d^NHS mental health dashboard (formerly the Five Year Forward View for Mental Health Dashboard)^19^ and Improving Access to Psychological Therapies (IAPT) Dashboard.^20^

The search identified 12 publicly available sources of health data within the perinatal period that might reasonably be expected to contain routine data on mental health. Seven data sources were excluded for not meeting the study eligibility criteria (Figure 1).

Five data sources (hosting four data sets) of publicly available routine data were included in the study, although one (iViewPlus) was decommissioned during our investigation. Table 1 presents the utility assessment of each data source. We found considerable limitations with the platforms and the data sets which made it impossible to obtain PMH condition prevalence rates using only the data presented in the platforms. Similarly, none of the data provided sociodemographic information which could be filtered or extracted to explore potential variance in PMH conditions by geographical area and/or by specific social determinants of health, such as ethnicity or socioeconomic status. The data sources and technical specifications for the data sets were often difficult to locate and supporting information (eg, for key definitions and data quality) was frequently insufficient (this information is presented in the Table 1 footnotes).

**TABLE 1:** A summary of the utility of publicly accessible data sources (platforms) of routine data for estimating the prevalence of PMH conditions and variation by sociodemographic factors.

Platform (and data)	Data period	Utility of the platform^a^	Utility of the data/limitations	PMH prevalence (Yes/No)	Variance in PMH prevalence by sociodemographics (Yes/No)
Community Services Data Set (CSDS) dashboard (using the CSDS)^24^	Version 1.5 available from October 2021– May 2022	Monthly summary statistics are provided via the dashboard. Data cannot be extracted for additional analysis using other software.	Contains health data from publicly funded community services, including health visiting services. The only PMH data item is the number of service referrals for ‘maternal mood problems’. A service referral is defined as ‘a request for a care service to be provided for a person needing care’. Information on the source of the referral is not provided (as it is for some other data items). The data cannot be filtered by region or PMH condition and it is unknown how many women may not have consented to referral (and therefore may be missing from the data). It is specified in the Public Health England (PHE) PMH Data Catalogue that a data item will be added to the CSDS for whether health visitors asked about PMH at a contact roughly 9 months following the birth^b^. This item does not currently appear in the data set. No sociodemographic (SD) data available.	No	No
Hospital Episode Statistics (HES) dashboard^25^	April 2008–September 2022	Annual summary statistics are provided. Monthly summaries are also available in Excel format of the number of inpatient care and outpatient appointments in specialist Perinatal Mental Health Services	Contains records of all patients admitted to NHS hospitals in England, including data from specialist Perinatal Mental Health Services. Gives the number of appointments offered and attended by the Perinatal Mental Health Service and the number of admissions into the service. Cannot be filtered by region, PMH condition or to ascertain the number of women accessing appointments (as some will have had more than one appointment). This data would produce an underestimation of PMH prevalence as it only contains women who actively engaged with services and had severe/acute illness necessitating their engagement. No SD data available.	No	No
iViewPlus (using the MSDS)^26^ – Platform decommissioned and unavailable from July 2022	Apr 2015‐July 2018^c^	Annual summary statistics are provided. Data cannot be extracted for analysis using other software.	Included limited data from the MSDS, booking appointment data only – one explicit PMH data item included, ‘PMH prediction and detection’ defined: ‘as identified at the Booking Appointment, whether or not the recommended questions for prediction and detection of mental health issues were asked’^d^, and if previous mental health conditions were identified. It did not reflect if only one of the two aspects were discussed in the booking appointment and did not provide the outcomes so cannot indicate prevalence. SD data are available and can be cross‐tabulated (including by area) with other data items.	No	No
Mental Health Services dashboard (using the Mental Health Services Data Set; MHSDS)^27^	April 2016‐March 2020	Monthly and annual summary statistics are provided. Data cannot be extracted for additional analysis using other software.	Data captured by secondary mental health services (and depositing in the MHSDS) is linked to the MSDS to restrict the population to perinatal women. A view of the perinatal period is available. This gives an indication of how many women access services for clinically significant poor PMH from which we can produce an estimate (but only for women who are referred and take up care from secondary care services), and population estimates (the denominator – the number of birthing women in the time period) are not provided from which prevalence estimates can be produced. Spreadsheets for the number of people in the perinatal period are available for the SD factors: index of multiple deprivation (IMD), ethnicity and age. Cross‐tabulation of the number of women accessing services by these factors are presented but are not available by geographical area and the impact on prevalence of a number (intersectionality) of SD factors cannot be explored.	Yes (if denominator values were available)	Yes (if denominator values were available)
National Maternity dashboard (NMD; using the MSDS and other data sets)^28^	Jan 2018 to Jan 2022	Annual NHS clinical quality improvement metrics and national maternity indicators are provided. Data cannot be extracted for analysis using other software.	Includes limited data from the Maternity Services Data Set (MSDS). The other data sources presented in the platform do not include PMH data (and are largely not routine data and): Clinical Quality Improvement Metrics, the National Maternity Indicators, and Continuity of Carer policy by NHS Trust, plus data from the National Maternity and Perinatal Audit, MBRRACE‐UK, CQC Maternity Survey, NHS Staff Survey and General Medical Council Doctors in Training Survey. None of the performance indicators either directly or indirectly include perinatal mental health (PMH) outcomes. Sociodemographic data are reported but cannot be cross‐tabulated with other data items.	No	No

^a^ The criteria used to audit the utility of sources of publicly available PMH data were: (1) accessibility; (2) data relevance; (3) technical specification. Appraisal of the first two utility criteria is included in the table, with the third criterion described here. Technical specification utility was not appraised if the data set/platform did not contain any PMH data items and is not included for the iViewPlus as this platform is no longer available. For the MHSDS Dashboard, the data requirements and details of updates to the data were easily accessible online. No issues of data quality are flagged in these resources. For the PMH Fingertips Dashboard, the technical specification is integrated into the platform so easy to find but the limitations of the data are not clearly stated and it is unclear whether the data only relates to the perinatal period and how this is defined.

^b^ The data which is due to be added is for the question: while you were pregnant or since your baby was born, did you experience any problems with your emotional or mental health or have a period of feeling low? Health Visitors provide a mandatory postnatal PMH check at 6‐8 weeks which includes asking about PMH, yet there are no plans to include the outcome of self‐reported emotional health recorded at this routine appointment in the CSDS.

^c^ The data from the NMD and iViewPlus cannot be combined to produce a dataset for the full period 2015 to 2021 as the platforms present different data items, with considerable variation in how the data are presented and how they can be explored.

^d^ The NICE guidance on managing PMH states that clinicians should consider using mental health identification measures at a woman's first contact with primary care, at the midwifery booking appointment, and during the early postnatal period.^17^ This should include both prediction and detection elements (asking about personal and family history of MH needs and assessment using the Whooley questions) and validated anxiety and depression assessment tools, with further assessment via the Patient Health Questionnaire 9 (PHQ‐9), Generalised Anxiety Disorder (GAD)‐2, GAD‐7 and Edinburgh Postnatal Depression Scale (EPDS) if there are concerns about PMH issues.^17^, ^29^, ^30^, ^31^, ^32^

For England, the primary source of perinatal data is the Maternity Services Data Set (MSDS; currently version 2.0).^33^ This provides data items captured at key time‐points during midwifery care, including the booking appointment (when the woman is 8‐12 weeks pregnant) and birth, and is presented by geographical area. It includes a mandatory data group *MSDS101PregnancyBooking* which data providers are required to share monthly with NHS Digital for every woman's pregnancy and birth (although only 80% of data are deposited with variability in the extent and consistency of data sharing across CCGs and NHS Trusts, to the extent that some NHS Trusts do not consistently share any data for some mandated items).

Two platforms are used to share the MSDS: the National Maternity Dataset (also known as the Maternity Services dashboard) and iViewPlus.^26^, ^28^ However, despite the mandatory data group, the National Maternity Dataset does not include any information on PMH outcomes, and the iViewPlus contains only one. This data item (referenced M101150) is called ‘PMH prediction and detection’ and is a binary composite item taken from the midwifery booking appointment and includes: whether the PMH screening tool (Whooley) was asked or not, and if previous mental health conditions were identified.^34^ The purpose of the Whooley questions is to screen for PMH concerns, so an indicator which shows whether the question is asked or not is not a useful measure of the presence/absence of a PMH concern.^29^ Whilst a history of previous mental ill‐health increases the risk of PMH, it does not predict it. Neither on their own, nor in combination, do these indicators enable any prevalence rates to be identified. In addition, the iViewPlus was decommissioned in July 2022 and no replacement platform has been announced. The annual NHS Maternity Statistics report which uses MSDS data does not include any PMH content,^35^ so now that the iViewPlus is unavailable there is no publicly available reporting for the item.

Through linkage of the MSDS and Mental Health Services Data Set (MHSDS, Version 5.0), the Mental Health Services dashboard provides a view of the data on secondary mental health service use for the perinatal period and is the only platform from which a prevalence estimate could potentially be produced.^27^, ^33^, ^36^ It provides information on the number of women (aged 16 or over) who received a mental health referral to a secondary care NHS trust in the periods between their pregnancy booking appointment and 12 (*n* = 770) or 24 months (*n* = 945) post‐pregnancy. There is also data available on variation by ethnicity and socioeconomic status. However, the MHSDS only provides information on women who are referred and take up care from secondary care services, so is only indicative of moderate to severe and complex PMH conditions. As such it is likely to underestimate PMH need. In addition, this dataset does not provide information on the required denominator values (the total number of women who had a pregnancy and were within 12–24 months of the birth) meaning that prevalence estimates cannot be produced. Likewise, whilst information on the ethnicity and socioeconomic status of the women who received referral to secondary care services are provided for the different time periods, denominator values for these groups are not provided so variation by group and by geographical area cannot be calculated.

Information reviewed in the Public Health England PMH Data Catalogue indicated that there may be plans in place to improve PMH data collection by NHS Digital, both in the MSDS and CSDS. In the latest version of the MSDS technical output specification (version 2.0, published 17 November 2022) a data group for PMH assessment scales was specified (*MSD601AnonSelfAssessment*: including the Edinburgh Postnatal Depression Scale, Generalised Anxiety and Depression (GAD‐7), Patient Health Questionnaire (PHQ9) and Whooley questions; Generalised Anxiety Disorder (GAD)‐2 is not included).^29^, ^30^, ^31^, ^32^ However, this data cannot yet be accepted by NHS Digital: ‘there are currently no assessment tools in scope for MSD601 Anonymous Self‐Assessment. Any records submitted in this table will be rejected.’ We have been unable to find any information on when mandatory sharing of data on PMH assessment scales will occur. Likewise, it is specified that a data item will be added to the CSDS for whether health visitors asked about PMH at a contact roughly 9 months following the birth (‘while you were pregnant or since your baby was born, did you experience any problems with your emotional or mental health or have a period of feeling low?’).^22^ However, there is no information in the data sets or supporting documentation on when mandatory sharing and reporting of these data items will occur, whether the reporting relates to if the question was asked and/or the answer (outcome), and if reporting will be public, for example, will these data items be included in the National Maternity Dataset in future.

## 5 DISCUSSION

We have identified a PMH data ‘blind spot’ in open‐access routine health datasets. There are a number of inadequacies in the platforms, source data, and accompanying technical specifications which make it impossible to provide information on the prevalence of poor PMH in England and understand variation by geographical area and social determinants of health. As stated in the Department of Health and Social Care 2022 independent report on using health data for research and analysis, ‘data are at the core of all good work in healthcare’ but ‘raw data does not do great work on its own. This data must be curated, managed, cleaned, reshaped and prepared by people. Then it must be made available in well‐designed platforms, which earn public trust through security and transparency, and which facilitate sharing and re‐use of prior work’.^37^ The MSDS currently reports on an uninformative composite indicator which does not identify the prevalence of mental ill‐health. There are no publicly available datasets which report on health visitor or GP assessments of PMH, and certainly no dataset that links these three services who are integral to the detection of PMH conditions. Limited information is available about the number of referrals into PMH secondary care services, but with no population‐level data to enable prevalence to be calculated. These issues highlight why PMH prevalence rates continue to be estimated from the PMH Fingertips information. However, this information does not provide reliable estimates that reflect area‐based sociodemographic variation, and as such cannot be used to plan services that meet local needs.

To address the data blind spot, greater linkage of data sets across services is needed with improved technical specification and data systems that capture the required information. There is recognition that gaps in service provision and unwarranted geographical variation cannot be tackled without high‐quality interoperable data analysed at local geographical area levels.^38^ Since 2014, NHS England have been pushing for interoperability across health and care systems, for integrated software and local organisation commitments to data quality and sharing.^39^ For this, all data collection sites need to have the infrastructure to analyse and validate the information prior to submission.

The MSDS was developed to drive achieving better care outcomes for mothers and babies by providing comparative mother and child‐centric data that could be used to improve clinical quality and service efficiency.^40^ The data are monitored against the MSDS specification to inform the commissioning of services to improve health and reduce inequalities and be valuable for local and national monitoring and research. The MSDS information page states that the data set ‘provides reliable information for local and national monitoring, reporting for effective commission, monitoring outcomes and addressing health inequalities’.^33^ However, we have shown that neither the MSDS nor any other publicly available data sources provide a routine data measure of the prevalence of poor PMH. Data on PMH screening and assessment is known to be completed by midwives and health visitors throughout the perinatal period as stipulated by NICE guidance.^17^ However, this data is not collated or shared in a meaningful way. If this data was captured systematically on local data systems and were included as key items within the MSDS (alongside key information on geographical location, ethnicity and socioeconomic circumstances), it would be possible to provide real‐time prevalence of PMH conditions at an area level.

In Bradford, a data collection pathway has been established within the health visiting service so that the outcomes of PMH screening questions (rather than whether the question was asked or not) and follow‐on assessment outcomes are routinely collected showing that it is possible to capture and report on this information within health systems.^41^ The same pathway implemented in maternity services and health visiting services across England, alongside an updated requirement by NHS Digital to include this information in the MSDS and CSDS, would enable PMH prevalence to be identified. This publicly available data set would be highly valuable for PMH research and service planning.^23^

Further, without PMH data linked to sociodemographic data, accurate PMH prevalence estimates that reflect area sociodemographic variation cannot be produced. Without this service providers cannot design and provide appropriate and responsive PMH services to support the needs of their maternal populations. Prady et al.^7^ quantified the scale of the disparities in the identification of PMH needs between white British/English speaking women and ethnic minority and women who do not speak English. There was very little research on the association between other dimensions of disadvantage (eg, area deprivation, education, occupation, disability, social capital or personal characteristics such as parity and marital/partnership status) and disparities in PMH detection despite the relationship between these factors and poor mental health being well‐established.^42^ The Office for Health Improvement and Disparities cite a lack of evidence of the association between social determinants of health and PMH difficulties as the reason for the prevalence estimates in PMH Fingertips not taking account of sociodemographic differences. They add that ‘we are not aware of any data or research on exactly how maternal mental health differs by socioeconomic status that would allow us to take this into account in our estimates but appreciate that this would be useful if possible in the future’.^9^ Whilst there is some evidence, there is quite clearly a need for research in this area.

One challenge to building this evidence base is the well‐known issue of poorly captured information on ethnicity and other protected characteristics, such as gender and sexuality, in health data.^43^ Researchers and service providers and commissioners require improved access to more useful and clearly specified PMH and sociodemographic data, which can then be used to generate the evidence that is needed to produce area estimates of PMH difficulties based on local variation in sociodemographic factors.

Based on our findings, we make the following recommendations to improve the utility of publicly available routine PMH data in England, with which accurate area‐based estimates of the prevalence of PMH difficulties can be made and therefore available for research and PMH service planning.

1. Changes to the maternity and community services routine health record systems to ensure that there is mandated recording of (a) the *outcomes* of routine screening and assessments of PMH conditions, and any referrals made for these conditions; and (b) inequality characteristics (eg, ethnicity, socioeconomic status, sexuality, etc.)., at all stages of the PMH pathway.
2. The above data are shared as a part of the requirements to existing for open‐access routine datasets (eg, MSDS, CSDS).
3. Enhance the interoperability of all PMH NHS data systems (eg, midwifery, health visiting and general practitioners) so that information on PMH (and other areas of concern/need) can be consistently collected and collated across the healthcare pathway into a single data source.
4. Inclusion of all perinatal data (including PMH assessment outcome data items and sociodemographic information) in one publicly available platform and with a data extraction function so further analysis outside the platform can be performed.
5. For the above publicly available platform to include PMH prevalence estimates adjusted for area‐based sociodemographic differences in PMH and routine inclusion of population reference values to make the data more comparable between geographical areas.
6. Clear and comprehensive descriptions of data items and statements of data limitations, which are easy to find and signposted from within data sources, including greater transparency in the process of data collection, how data items were derived and the limitations of the data.

These recommended changes would require adaptations to existing data systems at likely significant costs. However, there would be no costs incurred directly to practice as the data is already collected and recorded by practitioners, and if achieved, these changes would improve the chances of high quality, joined up clinical care for mothers and babies, thereby preventing longer‐term health and large cost consequences.

If these recommendations cannot be achieved, then other sources of accurate PMH prevalence estimates need to be identified. This could be through the use of research and/or health data available via data linkage and request – for example, the Clinical Practice Research Datalink (CPRD) combined with ONS socioeconomic data has recently been used by others to explore childhood exposure to poor maternal mental health.^44^ An alternative option would be to consider the use of other existing population surveys, such as the Adult Psychiatric Morbidity survey,^45^ ensuring they reach a representative sample of perinatal women.

## 6 CONCLUSION

There are currently no publicly available routine data sets which allow NHS services in England to identify the prevalence of PMH conditions, nor is there currently adequate data from which these prevalence estimates could be produced. We found inadequacies with the platforms, the data and the technical specification which made it challenging to interpret the data and impossible to produce PMH prevalence. The PMH Fingertips dashboard does not provide a satisfactory alternative as there are significant limitations with the estimation approach, especially the absence of adjustment for area‐based sociodemographic variation. We have made recommendations to improve the utility of publicly available routine PMH data with which accurate area‐based estimates of the prevalence of PMH difficulties can be made to improve PMH service planning and research activities.

## AUTHOR CONTRIBUTIONS

Sarah Masefield led the study and analysed the data. Josie Dickerson was principal investigator of the wider programme research. Josie Dickerson, Kathryn Willan, Zoe Darwin and Chandani Nekitsing contributed to study design. Sarah Masefield, Kathryn Willan and Josie Dickerson produced the first draft of the manuscript. All authors contributed to the manuscript revision and read and approved the submitted version.

## FUNDING INFORMATION

West Yorkshire and Harrogate Health and Care Partnership, NHS Wakefield Clinical Commissioning Group, £225,000. Sarah Blower and Josie Dickerson are also supported by National Institute for Health Research; Applied Research Collaboration – Yorkshire and Humber (NIHR; ARC‐YH; Ref: NIHR200166). The funding body was not involved in the design of the study and collection, analysis, and interpretation of data or in writing the manuscript.

## CONFLICT OF INTEREST STATEMENT

The authors declare that they have no competing interests.

## Data Availability

The data sources analysed during the current study are available in the repositories: Community Services Data Set Dashboard: https://digital.nhs.uk/data‐and‐information/data‐collections‐and‐data‐sets/data‐sets/community‐services‐data‐set/community‐services‐data‐set‐supplementary‐reports#prototype‐monthly‐dashboard
Hospital Episode Statistics (HES) Dashboard: https://digital.nhs.uk/data‐and‐information/publications/statistical/provisional‐monthly‐hospital‐episode‐statistics‐for‐admitted‐patient‐care‐outpatient‐and‐accident‐and‐emergency‐data/april‐2022‐‐‐september‐2022
iViewPlus (Platform decommissioned): https://digital.nhs.uk/services/iview-and-iviewplus
Mental Health Services Dashboard: https://digital.nhs.uk/data-and-information/data-collections-and-data-sets/data-sets/mental-health-services-data-set
National Maternity Dashboard: https://digital.nhs.uk/data‐and‐information/data‐collections‐and‐data‐sets/data‐sets/maternity‐services‐data‐set/maternity‐services‐dashboard
Public Health England Fingertips: https://fingertips.phe.org.uk/profile-group/mental-health/profile/perinatal-mental-health
